# Determinants of access to experimental antiretroviral drugs in an Italian cohort of patients with HIV: a multilevel analysis

**DOI:** 10.1186/1472-6963-12-38

**Published:** 2012-02-15

**Authors:** Enrico Girardi, Paola Scognamiglio, Claudio Angeletti, Andrea Gori, Dora Buonfrate, Massimo Arlotti, Giovanni Mazzarello, Antonella Castagna, Massimo Andreoni, Antonella d'Arminio Monforte, Andrea Antinori, Giuseppe Ippolito

**Affiliations:** 1 Department of Epidemiology and Preclinical Research, National Institute for Infectious Diseases "L. Spallanzani", IRCCS, Via Portuense 292, 00149, Rome, Italy; 2 Infectious Diseases Unit, San Gerardo Hospital, Milano-Bicocca University, Via Pergolesi 33, 20900, Monza, Italy; 3 Infectious and Tropical Diseases Unit, Azienda ULSS6-Vicenza Hospital, Via F.Rodolfi 37, 36100, Vicenza, Italy; 4 Department of Internal Medicine 1 - Infectious Disease Unit, Infermi Hospital, Via Settembrini 2, 47900, Rimini, Italy; 5 Clinic of Infectious Diseases, University of Genoa, San Martino Hospital, Largo Rosanna Benzi 10, 16132, Genoa, Italy; 6 Department of Infectious Diseases, San Raffaele Scientific Institute, Via Olgettina 60, 20132, Milan, Italy; 7 Clinic of Infectious Diseases, Tor Vergata University, Viale Oxford 81, 00133, Rome, Italy; 8 Clinic of Infectious Diseases, S. Paolo Hospital, University of Milan, Via A Di Rudini 8, 21142, Milan, Italy; 9 Clinical Department, National Institute for Infectious Diseases "L. Spallanzani", IRCCS, Via Portuense 292, 00149, Rome, Italy; 10 Members of the I.Co.Na Foundation Study are listed in an appendix

**Keywords:** antiretroviral therapy, experimental drug, HIV, cohort study, clinical trial, expanded access program, multilevel analysis

## Abstract

**Background:**

Identification of the determinants of access to investigational drugs is important to promote equity and scientific validity in clinical research. We aimed to analyze factors associated with the use of experimental antiretrovirals in Italy.

**Methods:**

We studied participants in the Italian Cohort of Antiretroviral-Naive Patients (ICoNA). All patients 18 years or older who had started cART (≥ 3 drugs including at least two NRTI) after their enrolment and during 1997-2007 were included in this analysis. We performed a random effect logistic regression analysis to take into account clustering observations within clinical units. The outcome variable was the use of an experimental antiretroviral, defined as an antiretroviral started before commercial availability, in any episode of therapy initiation/change. Use of an experimental antiretroviral obtained through a clinical trial or an expanded access program (EAP) was also analyzed separately.

**Results:**

A total of 9,441 episodes of therapy initiation/change were analyzed in 3,752 patients. 392 episodes (360 patients) involved an experimental antiretroviral. In multivariable analysis, factors associated with the overall use of experimental antiretrovirals were: number of experienced drugs (≥ 8 drugs versus "naive": adjusted odds ratio [AOR] = 3.71) or failed antiretrovirals(3-4 drugs and ≥ 5 drugs versus 0-2 drugs: AOR = 1.42 and 2.38 respectively); calendar year (AOR = 0.80 per year) and plasma HIV-RNA copies/ml at therapy change (≥ 4 log versus < 2 log: AOR = 1.55). The probability of taking an experimental antiretroviral through a trial was significantly lower for patients suffering from liver co-morbidity(AOR = 0.65) and for those who experienced 3-4 drugs (vs naive) (AOR = 0.55), while it increased for multi-treated patients(AOR = 2.60). The probability to start an experimental antiretroviral trough an EAP progressively increased with the increasing number of experienced and of failed drugs and also increased for patients with liver co-morbidity (AOR = 1.44; p = 0.053). and for male homosexuals (vs heterosexuals: AOR = 1.67). Variability of the random effect associated to clinical units was statistically significant (p < 0.001) although no association was found with specific characteristics of clinical unit examined.

**Conclusions:**

Among patients with HIV infection in Italy, access to experimental antiretrovirals seems to be influenced mainly by exhaustion of treatment options and not by socio-demographic factors.

## Background

Removing the barriers to accessing experimental drugs is an important goal from both a scientific and ethical point of view. In fact, generalizable therapeutic research requires a study population that is representative of the population that will eventually be in need of the drug being studied [1,2]. Moreover, for patients with serious or life-threatening diseases who cannot be treated effectively with approved drugs, access to experimental drugs may represent the only possible way of receiving effective treatment [3].

The issue of accessing experimental drugs has been debated for persons with HIV infection since the first decade of the epidemic. When the first antiretrovirals entered clinical trials, in response to the need of patients for whom no effective treatment was available, the US issued a new regulation that allowed experimental drugs to be distributed outside clinical trials in the context of so-called Expanded access programs (EAP) [4]. Subsequently, similar regulations were issued in other countries [5].

Since zidovudine was first approved for clinical use, the possibility of treating HIV infection has dramatically expanded and more than 20 drugs are now available in industrialized countries [6]. Nonetheless, there is still a sizeable population of patients who experience virological failure to the three main classes of antiretrovirals and need access to new drugs [7,8].

Knowledge of patterns of access to experimental drugs by persons with HIV is still incomplete. A number of studies have analyzed the barriers that prevent access to clinical trials of antiretroviral drugs. Cross-sectional surveys on HIV patients conducted in North America found that ethnic minorities, women and patients with public or no health care insurance were underrepresented in clinical trials [9-14]. In European studies, the mode of HIV transmission and socio- economic status were reported to be associated with participation in clinical trials [15,16]. On the other hand, little information is available on the overall access to experimental drugs (through clinical trials or EAPs). A survey conducted on a probability sample of US patients with HIV found that ethnic minorities and persons cared for in private health maintenance organizations had a lower probability of receiving experimental drugs [17].

The aim of our study was to analyze the access to experimental antiretrovirals and the variation over time in the context of a national health service providing universal access to antiretroviral treatment. To this end we analyzed data recorded between 1997 and 2007 in a multicentre cohort study in Italy conducted on patients with HIV infection who were antiretroviral naïve at the time of enrolment [18].

## Methods

This analysis was conducted within the Italian Cohort of Antiretroviral-Naive Patients (ICoNA) study, an observational cohort of HIV-infected individuals who were antiretroviral naïve at enrolment [18]. This cohort was set up in January, 1997 and to date consists of more than 7,000 patients from 50 infectious disease units in Italy. Initiation and discontinuation dates of each antiretroviral drug, HIV-viral load and CD4 cell count at each clinical visit (every 4-6 months on an average) were recorded for each enrolled patient.

### Ethics statement

All individuals signed an informed consent prior to enrolment and the study was approved by the Ethics Committee of each participating institution that are listed in an appendix.

All patients 18 years or older who had started cART (≥ 3 drugs including at least two NRTI) during 1997-2007 were included in this analysis.

For each subject included in the analysis we analyzed the "episodes of therapy initiation/change", defined as any initiation of a single antiretroviral drug as part of the initial or subsequent regimen.

The initiation of a co-formulated drug without change in the component medicaments of the regimen was considered as a therapy change. On the other hand, change of therapeutic regimen, consisting of discontinuation of a drug without substitution, is not included in this definition and we did not consider consecutive prescriptions of the same antiretroviral separated by discontinuation. A CD4 count and a viral load determination had to be available within 6 months before and 15 days after therapy initiation/change for the episode to be included in the analysis.

We speculated that for physician and patient, there is a chance of starting an experimental antiretroviral when a new regimen is started or a new drug is introduced in a cART combination. For the purpose of this analysis, we defined an experimental antiretroviral as any antiretroviral drug started when it was not yet licensed to be legally marketed and sold in Italy (Table 1). If participation in a clinical trial was not recorded and the experimental antiretroviral was started after the first ethical approval of an EAP for that drug in Italy, we assumed that the drug was obtained through an EAP; otherwise the experimental antiretroviral was considered to be obtained through a clinical trial.

**Table 1:** Year of regulatory approval in Italy of antiretroviral drugs, 1987-2008

Year	NRTI	NNRTI	PI	EI	II
1987	Zidovudine (AZT)				

1992	Didanosine				

1995	Zalcitabine				

1996	Stavudine Lamivudine (3TC)		Indinavir Ritonavir Saquinavir HG		

1998	AZT/3TC Co-formulation	Nevirapine	Nelfinavir		

1999	Abacavir (ABC)	Efavirenz	Saquinavir SG		

2001	ABC/AZT/3TC Co-formulation		Amprenavir Lopinavir/Ritonavir		

2002	Tenofovir (TDF)				

2004			Atazanavir	Enfuvirtide	

2005	Emtricitabine (FTC) TDF/FTC Co-formulation ABC/3TC Co-formulation		Fosamprenavir Tripanavir		

2007			Darunavir^a^		

2008				Maraviroc^a^	Raltegravir^a^

Not approved		Delavirdine Atevirdine DP 083			

NRTI = Nucleoside Reverse Transcriptase Inhibitor; NNRTI = Non-nucleoside Reverse Transcriptase Inhibitor; PI = protease inhibitors; EI = Entry

and Fusion Inhibitors; II = Integrase Inhibitor

^a ^Not used in the IcoNa cohort prior to regulatory approval

### Statistical analysis

Descriptive statistical techniques were used to provide a general profile of the study population.

The temporal trend in the use of experimental antiretroviral was evaluated by plotting the proportion of therapy initiation/change by calendar year.

We computed the proportion of therapy initiation/change with an experimental antiretroviral through a trial and an EAP by the clinical centre and we evaluated the association between them by using Pearson's correlation coefficient.

Univariable association between the distribution of episodes of therapy initiation/change according to the presence of an experimental antiretroviral and selected characteristics were tested using a Pearson's chi-square test stratified by clinical centre [19].

#### Outcome variables

Multivariable logistic models were performed to assess adjusted odds ratios (AOR) for initiation of an experimental antiretroviral. In model 1 the outcome variable was the overall use of experimental antiretroviral drugs, regardless of whether the drug was obtained through a clinical trial or an EAP. In model 2 the outcome variable was the use of an experimental antiretroviral obtained through a trial, and in model 3 the outcome variable was the use of an experimental antiretroviral obtained through an EAP.

Correlation between statistical units coming from the same clinical centre was taken into account by adding a random intercept to the models. We did not take into account clustering observations within patients since this outcome was a rare event (4%), and clusters without events would have been dropped by the analysis [20].

The covariates introduced in the models concerned the characteristics of clinical centres (categorized as hospital vs. university/research centre and number of reported AIDS cases), demographic, epidemiological (gender, age at therapy change, country of origin, occupation, marital status, years of education, HIV exposure category) and clinical characteristics of the subjects (clinical stage of HIV disease, CD4 cell count, plasma HIV-RNA, presence of cardio-vascular/metabolic diseases or neoplasm, presence of liver comorbitidy (liver diseases and/or HBsAg positivity and/or anti-HCV positivity) at the time of therapy change.

We also analyzed variables indicating exhaustion of treatment options, by considering the number of experienced antiretroviral drugs and of virologically failed antiretroviral drugs (defined as plasma HIV-RNA above 500 copies per mL after more than 4 months of continuous use of the drug) [21]. In order to take into account the different availability over time of experimental antiretrovirals, we also adjusted the estimates for the calendar year of antiretroviral prescription.

Analyses were performed with Stata Statistical Software: Release 10 (Stata Corp, College Station, Tex).

## Results

### Patients

**Figure 1:**
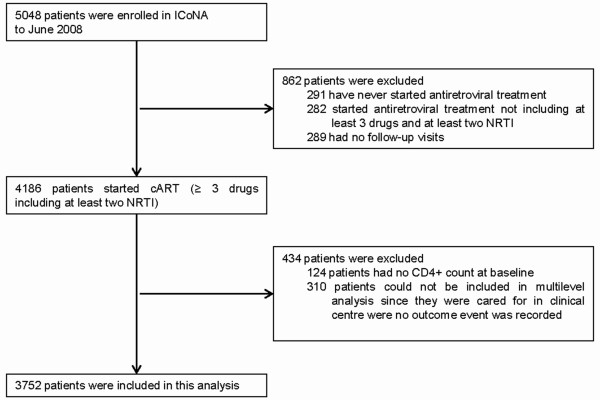
**Flow diagram of patients included in this analysis**.

3,752 patients were included in our analysis (Figure 1). The median age at enrolment was 37 years (inter-quartile range [IQR]: 32.7-42.5). Women accounted for 29.6% of the total study population. The majority of patients were born in Italy (92.4%). With regard to HIV exposure category, individuals infected through heterosexual contacts accounted for 38.5%, through homosexual contacts for 21.4%, and those infected through injecting drug use for 33.6% of the total.

The median CD4 cell count at baseline was 279/mm^3 ^(IQR: 139-429.5); HIV-viral load at baseline (log 10 copies/ml) was 4.73 (IQR: 4.07-5.23). Clinically defined AIDS was present at enrolment in 16.9% of patients, while liver comorbitidy was present in 40.8%. 45.6% of patients were cared for in a teaching hospitals.

### Episodes of therapy initiation/change

A total of 9,441 episodes of therapy initiation/change were analyzed of which 392 episodes, related to 360 patients (9.6% of total), involved an experimental antiretroviral. In 192 episodes (in 188 patients, 5.0% of the total) the experimental drug was obtained through a trial, and in 200 (in 172 patients, 4.6% of the total) through an EAP.

**Figure 2:**
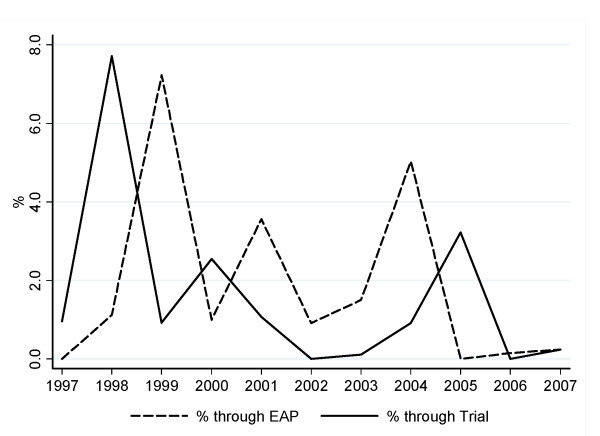
**Proportion of regimen initiation/changes involving an experimental antiretroviral by calendar year**.

The trend in experimental antiretroviral use during the study period is shown in Figure 2; this trend partly reflects the availability of clinical trials or EAPs over time in Italy (data not shown). The proportion of episodes of therapy initiation/change involving the use of an experimental antiretroviral obtained through a trial varied markedly in different years and peaked in 1998 at 7.7%. A marked variability was also observed for a proportion of episodes of therapy initiation/change involving the use of an experimental antiretroviral obtained through an EAP which peaked (7.2%) in 1999.

**Figure 3:**
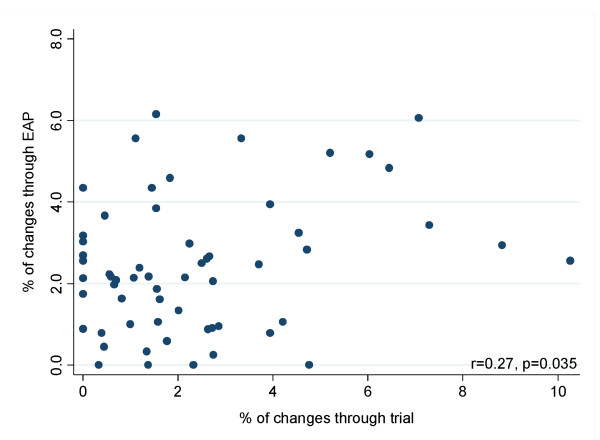
**Regimen changes involving an experimental antiretroviral through clinical trials or expanded access programs (EAP) in each clinical centre**.

The proportion of therapy initiation/change with an experimental antiretroviral (Figure 3), ranges between 0.0 and 10.3% for trials and 0.0 and 6.2% for EAPs in different clinical centres. There was a significant correlation (r = 0.27, p = 0.04) between the proportion of regimen changes/initiation with an experimental antiretroviral obtained through a trial and through an EAP by clinical centre.

Table 2 shows distribution of episodes of therapy initiation/change according to the presence of an experimental antiretroviral, by socio-demographic, behavioural and clinical characteristics of the patients and characteristics of the clinical centre. In univariable analysis, the characteristics of the clinical centre where the patient was cared for, viral load, presence of liver comorbidities and previous antiretroviral treatment at the time of therapy change were associated with probability of receiving an experimental antiretroviral.

**Table 2:** Use of experimental antiretroviral drugs in a therapy initiation/change episode by demographic/epidemiological characteristics of patients and clinical and laboratory data at the time antiretroviral treatment (ART) initiation/change

	Therapy initiation/change episodes including an experimental antiretroviral		Total therapy initiation/change episodes	p-value
	In clinical trial N (%)	**In EAP **^a^ N (%)		
**Total reported AIDS cases in the clinical centre**				0.02
0-250	34 (2.2)	34 (2.2)	1.575	
251-500	54 (2.7)	46 (2.3)	2.004	
501-1000	54 (2.4)	42 (1.9)	2.212	
> 1000	50 (1.4)	78 (2.1)	3.650	
**Type of clinical centre**				0.05
Non-teaching hospital	113 (2.3)	118 (2.4)	4.993	
Teaching hospital	79 (1.8)	82 (1.8)	4.448	
**Gender**				0.80
Females	56 (1.9)	58 (2.0)	2.889	
Males	136 (2.1)	142 (2.2)	6.552	
**Age at ART initiation/change**				0.48
18-34	67 (2.5)	56 (2.1)	2.687	
35-39	48 (1.8)	60 (2.3)	2.609	
40-44	40 (2.0)	37 (1.9)	1.960	
> = 45	37 (1.7)	47 (2.2)	2.185	
**Place of birth**				0.36
Italy	178 (2.0)	191 (2.2)	8.774	
Other countries	14 (2.1)	9 (1.3)	667	
**Years of education**				0.22
< = 8	78 (1.7)	90 (1.9)	4.624	
9-13	55 (2.2)	56 (2.2)	2.530	
> 13	10 (2.3)	11 (2.5)	432	
Unknown	49 (2.6)	43 (2.3)	1.855	
**Marital status**				0.29
Never married	93 (1.9)	94 (1.9)	4.864	
Married	99 (2.2)	106 (2.3)	4.577	
**Occupational status**				0.53
Unemployed	47 (2.0)	47 (2.0)	2.345	
Employed	138 (2.1)	145 (2.2)	6.675	
Other	7 (1.7)	8 (1.9)	421	
**HIV exposure category**				0.08
Heterosexual	73 (2.0)	68 (1.9)	3.597	
Homosexual/Bisexual	48 (2.4)	58 (2.9)	2.010	
IDU (Active+Former)	56 (1.7)	65 (2.0)	3.237	
Other/Unknown	15 (2.5)	9 (1.5)	597	
**Nadir CD4 cell count (cells/ml)**				0.07
< 200	86 (2.0)	104 (2.4)	4348	
200-349	51 (1.7)	51 (1.7)	2965	
> = 350	55 (2.6)	45 (2.1)	2128	
***Clinical and laboratory data at treatment initiation/change***				
**Clinical AIDS**				0.10
*No*	161 (2.1)	151 (2.0)	7.600	
Yes	31 (1.7)	49 (2.7)	1.841	
**CD4 cell count (cells/ml)**				0.10
< 200	64 (2.7)	56 (2.3)	2387	
200-349	45 (1.8)	49 (2.0)	2444	
> = 350	83 (1.8)	95 (2.1)	4610	
**HIV RNA (log 10 copies/ml)**				< 0.001
< 2	22 (1.0)	31 (1.4)	2275	
2-3	48 (2.0)	53 (2.3)	2345	
> = 4	122 (2.5)	116 (2.4)	4812	
**Presence of hepatic comorbidities**				0.01
No	127 (2.4)	103 (1.9)	5313	
Yes	65 (1.6)	97 (2.3)	4128	
**Presence of other ^b ^comorbidities**				0.15
No	179 (2.1)	177 (2.1)	8384	
Yes	13 (1.2)	23 (2.2)	1057	
**Number of experienced drugs**				< 0.001
Naive	117 (3.2)	42 (1.2)	3.647	
3-4	44 (1.2)	74 (2.1)	3.524	
5-7	15 (0.9)	50 (3.0)	1.675	
> = 8	16 (2.7)	34 (5.7)	595	
**Number of failed drugs**				< 0.001
0-2	153 (2.3)	94 (1.4)	6.727	
3-4	26 (1.3)	62 (3.3)	2.047	
> = 5	13 (1.9)	44 (6.6)	667	

a Expanded access program; b cardio-vascular/metabolic diseases or neoplasm

In multivariable logistic regression (Table 3), the probability of receiving an experimental antiretroviral through a trial or an EAP (model 1) decreased significantly over time (adjusted odds ratio [AOR] = 0.80 per year; p < 0.001). The overall probability of taking an experimental antiretroviral (model 1) increased for those who had experienced a higher number of antiretrovirals (≥ 8 drugs versus "naive": AOR = 3.71; p < 0.001) or who had failed a higher number of antiretrovirals (3-4 drugs and ≥ 5 drugs versus 0-2 drugs: AOR = 1.42, p = 0.03, and 2.38, p < 0.001, respectively) and for those who had an higher HIV viral load at regimen change (≥ 4 log copies HIV RNA/ml versus < 2 log copies HIV RNA/ml: AOR = 1.55, p = 0.02).

**Table 3:** Adjusted odds-ratios (AOR) of the use of experimental antiretroviral drugs in a therapy initiation/change episode by demographic/epidemiological characteristics of patients and clinical and laboratory data at the time of initiation/change

	Model 1			Model 2			Model 3		
	Overall use of an experimental antiretroviral			Use of an experimental antiretroviral in a clinical trial			**Use of an experimental antiretroviral in an EAP**^a^		
	AOR	95%CI	*p*	AOR	95%CI	*p*	AOR	95%CI	*p*
**Total AIDS cases reported in the clinical centre**			0.94			0.79			0.93
0-250	1.00			1.00			1.00		
251-500	1.10	(0.64-1.91)		1.07	(0.50-2.29)		1.07	(0.58-1.96)	
501-1000	1.03	(0.59-1.79)		0.97	(0.45-2.11)		0.99	(0.54-1.81)	
> 1000	0.93	(0.54-1.60)		0.74	(0.35-1.59)		1.18	(0.65-2.11)	
**Type of clinical center**			0.77			0.82			0.37
Non-teaching hospital	1.00			1.00			1.00		
Teaching hospital	0.94	(0.62-1.42)		1.07	(0.61-1.88)		0.82	(0.53-1.27)	
**Gender**			0.41			0.69			0.48
Female	1.00			1.00			1.00		
Male	0.89	(0.67-1.18)		0.92	(0.62-1.37)		0.87	(0.59-1.28)	
**Age at therapy initiation/change (per 5 year increase)**	1.00	(0.94-1.08)	0.90	0.99	(0.90-1.09)	0.82	1.02	(0.93-1.12)	0.70
**Place of birth**			0.36			0.97			0.14
Italy	1.00			1.00			1.00		
Other countries	0.80	(0.51-1.28)		1.01	(0.56-1.83)		0.57	(0.28-1.19)	
**Years of education**			0.64			0.76			0.81
< = 8	1.00			1.00			1.00		
9-13	1.14	(0.87-1.48)		1.19	(0.82-1.73)		1.11	(0.77-1.59)	
> 13	1.24	(0.75-2.05)		1.23	(0.60-2.50)		1.33	(0.67-2.64)	
Unknown	0.96	(0.70-1.32)		0.97	(0.62-1.52)		0.97	(0.63-1.49)	
**Marital status**			0.07			0.41			0.03
Never married	1.00			1.00			1.00		
Married	1.25	(0.99-1.58)		1.15	(0.83-1.59)		1.43	(1.03-1.98)	
**Occupational status**			0.79			0.92			0.81
Unemployed	1.00			1.00			1.00		
Employed	1.08	(0.83-1.41)		1.08	(0.74-1.57)		1.07	(0.74-1.55)	
Other	1.19	(0.65-2.17)		1.08	(0.46-2.53)		1.30	(0.57-2.97)	
**HIV exposure category**			0.09			0.99			0.01
Heterosexual	1.00			1.00			1.00		
Homosexual/Bisexual	1.33	(0.96-1.85)		1.05	(0.66-1.66)		1.67	(1.06-2.63)	
IDU (Active+Former)	0.85	(0.60-1.19)		1.00	(0.61-1.64)		0.79	(0.50-1.24)	
Other/Unknown	0.92	(0.57-1.47)		1.04	(0.57-1.88)		0.74	(0.35-1.55)	
***Clinical and laboratory data at treatment change***									
**Clinical AIDS**			0.81			0.12			0.24
No	1.00			1.00			1.00		
Yes	0.96	(0.72-1.29)		0.71	(0.45-1.10)		1.25	(0.86-1.81)	
**CD4 cell count (cells/ml)**			0.62			0.65			0.65
< 200	1.00			1.00			1.00		
200-349	0.89	(0.66-1.21)		0.82	(0.54-1.25)		0.97	(0.64-1.48)	
> = 350	1.02	(0.76-1.35)		0.89	(0.60-1.32)		1.15	(0.77-1.70)	
**HIV RNA (log 10 copies/ml)**			0.05			0.99			0.01
< 2	1.00			1.00			1.00		
2-3	1.27	(0.87-1.83)		1.05	(0.60-1.83)		1.39	(0.86-2.26)	
> = 4	1.55	(1.07-2.23)		1.02	(0.58-1.80)		2.05	(1.28-3.28)	
**Presence of hepatic comorbidities**			0.96			0.05			0.05
No	1.00			1.00			1.00		
Yes	1.01	(0.76-1.33)		0.65	(0.43-0.99)		1.44	(1.00-2.09)	
**Presence of other**^b ^**comorbidities**			0.85			0.98			0.96
No	1.00			1.00			1.00		
Yes	1.04	(0.71-1.52)		0.99	(0.54-1.83)		1.01	(0.62-1.64)	
**Number of experienced drugs**			< 0.001			< 0.001			< 0.001
Naive	1.00			1.00			1.00		
3-4	1.06	(0.78-1.44)		0.55	(0.35-0.87)		2.49	(1.58-3.93)	
5-7	1.40	(0.91-2.15)		0.57	(0.28-1.15)		3.54	(1.99-6.30)	
> 8	3.71	(2.13-6.45)		2.60	(1.08-6.28)		7.07	(3.43-14.56)	
**Number of failed drugs**			0.002			0.94			< 0.001
0-2	1.00			1.00			1.00		
3-4	1.42	(1.03-1.95)		1.07	(0.62-1.83)		1.57	(1.06-2.33)	
> 5	2.38	(1.47-3.86)		1.17	(0.48-2.83)		2.96	(1.67-5.23)	
**Calendar year (1 year increase)**	0.80	(0.76-0.84)	< 0.001	0.78	(0.73-0.85)	< 0.001	0.82	(0.76-0.88)	< 0.001
**Likelihood Ratio test for significance of Random effect**			< 0.001			< 0.001			0.001

a Expanded access program; b cardio-vascular/metabolic diseases or neoplasm

The probability of taking an experimental antiretroviral by participating in a clinical trial (model 2), was significantly lower for patients presenting a liver comorbidity (AOR = 0.65; p = 0.05) and for those who experienced 3-4 drugs compared to antiretroviral naive patients (AOR = 0.55; p = 0.010), while it increased for multi-treated patients (AOR = 2.60; p = 0.033). No significant association was found for the number of failed antiretrovirals and the viral load.

The probability of taking an experimental antiretroviral by participating in an EAP (model 3) was significantly higher for patients infected through homosexual contacts compared to those infected though heterosexual contacts and for those who were married. The probability of taking an experimental antiretroviral trough an EAP progressively increased with the increasing number of drugs experienced, with the increasing number of drugs failed and in those with a viral load at regimen change ≥ 4 log copies/ml. Patients presenting a liver co morbidity also had a higher probability of taking an experimental antiretroviral through an EAP (AOR = 1.44; p = 0.053).

In all three models, age, gender, place of birth, education, clinical stage (CD4 count or clinical AIDS), were not significantly associated with the probability of receiving an experimental antiretroviral. Similarly, no association was found with the characteristics of a clinical unit. On the other hand, the heterogeneity associated with the clinical centre, not explained by the covariates included in the model, was statistically significant in all three models (table 3).

## Discussion

Most of the published studies assessing access to experimental antiretroviral drugs were based on cross-sectional surveys on samples of persons with HIV [9,10,12,14,22,23]. We addressed this issue by analyzing data from a multi-centre cohort study over an 11-year period.

We found that almost 10% of patients received an experimental antiretroviral at least once. Marked variations were observed in different years of the study with an overall tendency of decreasing probability of receiving an experimental antiretroviral. Demographic and epidemiological characteristics of patients were not significant determinants of receiving experimental antiretrovirals. On the other hand, antiretroviral treatment experience was associated with access to experimental antiretrovirals both in trials and in EAP. Significant variability was associated with clinical centres, although no association was found with the specific characteristics of the clinical units that were examined in this analysis.

Previous studies have shown that persons with HIV have an high access to clinical trials of antiretrovirals. For example, surveys in the US found that 10-23% of patients interviewed reported experimental antiretroviral use [10,12,14,23]. These figures are generally higher than what is reported in our study. However, these studies were conducted mostly during the 1990s when a great number of new drugs were in registration studies [24] and when the patients had limited treatment opportunities and large expanded access programs were set up [25].

Our study also showed that 14.7% of patients who had treatment initiation/change in 1997-1999 had access to experimental antiretrovirals while this proportion decreased thereafter. However, in 2004-2007 the proportion of patients was still 3.3%, a figure which is higher than that reported for cancer patients [26].

Treatment history was an important determinant of accessing experimental antiretrovirals, however different patterns were observed for clinical trials and EAPs. The probability of receiving an experimental antiretroviral through a clinical trial was greater for naïve patients and when treatment was changed in patients who had experienced a higher number of drugs. This probably reflects the design of clinical trials that are mainly focused on naïve and multi-experienced patients [27]. On the other hand, the probability of using an experimental antiretroviral trough EAPs increased significantly for the number of drugs experienced and for those failed and for patients with. liver co-morbidities. Taken together, our results suggest that possibility of accessing experimental antiretrovirals has been and continues to be an important opportunity for patients with limited treatment options.

Previous studies suggested that access to experimental antiretrovirals may be lower in female patients [10,12], those with lower socio-economic status [17] and for ethnic minorities [12,14,17,23]. In our study, gender, occupational status and years of education were not associated with accessing experimental antiretrovirals. Foreign born patients had a somewhat lower, although not significantly, probability of receiving an experimental antiretroviral compared to patients born in Italy. However, foreign born patients constituted only 7.6% of our study population, so we may lack the power to detect significant association between ethnic origin and access to experimental drugs. In consideration of the increasing proportion of foreign born patients among those diagnosed with HIV/AIDS in Italy [28] as well as in other European countries [29] this aspect deserves close monitoring in the near future.

We observed a marked variability in access to experimental antiretrovirals in patients cared for in different clinical centres not captured by the covariates considered in our analysis. In a previous study, characteristics of the clinical unit providing care to patients with HIV, such as patients volume and proximity to an NIH-funded HIV trial centre, were independently associated with use of experimental drugs [17]. In our study no association was found with the specific characteristics of clinical unit examined, although, correlation between the probability of entering a clinical trial or an EAP in the same centres suggests the possible existence of common barriers to accessing experimental drugs. Previous studies have identified time constraints, lack of staff and training, concerns about new drugs as physicians as barriers to enrolling in clinical trials [30,31]. It remains to be determined whether such barriers had a role in determining differences among the clinical centres participating in this study.

It should be pointed out that our study was conducted on patients with HIV who were enrolled in a cohort study. Therefore, it can't be ruled out that they were more likely to be enrolled in clinical trials and EAPs than patients under care who were not in clinical studies and thus we could have overestimated the probability to access experimental antiretrovirals in Italy. On the other hand, it should be noted that the study population was recruited in a very large number of clinical centres in diverse geographic locations and with different characteristics, and that the distribution of our population by age, gender and mode of HIV acquisition is similar to that of AIDS cases reported in Italy during the study period [28].

In the analysis, we assumed that an experimental antiretroviral was obtained through an EAP if it was started after the first ethical approval in Italy of the program, and participation in a clinical trial was not recorded in the database. However, patients may have still be enrolled in a clinical trial when the EAP was active, so we may have misclassified some patients.

The study design also did not allow us to investigate the possible role of a series of patient factors such as fear of side-effects, distrust of researchers, concerns about research, and interference in everyday life which have been shown to represent significant barriers to accessing antiretroviral drug trials [32].

## Conclusions

In summary, our study shows that a substantial number of persons with HIV had the opportunity to access experimental antiretroviral drugs in our country and suggests that gender and socio-economic factors did not represent significant barriers to accessing these drugs. This is consistent with speculation that a national health system which provides universal access to care for persons with HIV, such as the Italian health system, may also reduce disparities in access to experimental drugs, in particular when the experimental drug represent an opportunity for patients with exhaustion of treatment options.

On the other hand, we also found that the chance of a patient of accessing experimental antiretroviral drugs varied widely in different clinical units: this is a potential source of non equity, and deserves further investigation. This finding also suggest the need of developing a national initiative aimed at facilitating the access of patients from different clinical centres to research on new drugs. In this context, establishing research networks among clinical centres and increasing the patient community information and involvement in clinical research may be useful tools.

## Competing interests

E.G. has received honoraria for presentation at workshops, or travel grants from Abbott, Bristol-Myers Squibb, Boheringer Ingelheim; A.G. received honoraria for presentation at workshops, or travel grants from Abbott, Bristol-Myers Squibb, Gilead, Pfizer, GlaxoSmithKline, ViiV Health Care; M.Ar. has received honorarium for expert opinion from Bristol-Myers Squibb and an unrestricted educational grant from Pfizer; M.An. has received honoraria for presentation at workshops, advisory board or research grants from Abbott, Bristol-Myers Squibb, Gilead, GlaxoSmithKline, Janssen-Cilag, Merck, Pfizer, ViiV HealthCare; A.d'A.M has received honoraria for presentation at workshops, advisory board or research grants form Abbott, Bristol-Myers Squibb, Janssen-Cilag, Pfizer, Gilead; A.A. has received honoraria for presentation at workshops, consultancy and advisory board, or research grants from Abbott, Bristol-Myers Squibb, Gilead, GlaxoSmithKline, Janssen-Cilag, Merck, Pfizer, ViiV HealthCare. A.C. has received honoraria for presentation at workshops, consultancy and advisory board, or research grants from Abbott, Boehringer-Inghelheim, Bristol Myers Squibb, Gilead Sciences, Glaxo-Smith Kline, Merck, Pfizer, Roche, and Tibotec (Johnson & Johnson). P.S., C.A.,G.M., D.B., G.I. declare that they have no conflict of interest.

## Authors' contributions

EG designed the study and drafted the manuscript. PS participated in the design of the study and drafted the manuscript. CA performed the statistical analysis and contributed with writing the manuscript. AG; DB; MA; GM; AC; A d'AM; AA; GI contributed with national coordination, data collection, and revised critically the manuscript. All authors have read and approved the final manuscript.

## Appendix - The Icona Foundation Study

### 1. GOVERNING BODY

M. Moroni (Chair), G. Angarano, A. Antinori, G. Carosi, R. Cauda, A. d'Arminio Monforte, G. Di Perri, M. Galli, F. Ghinelli, R. Iardino, G. Ippolito, A. Lazzarin, F. Mazzotta, C.F. Perno, P.L. Viale, F Von Schlosser.

### 2. SCIENTIFIC SECRETARY

A d'Arminio Monforte

### 3. STEERING COMMITTEE

A. Ammassari, A. Antinori, C. Balotta, P. Bonfanti, M.R. Capobianchi, A. Castagna, F. Ceccherini-Silberstein, A. Cozzi-Lepri, A. d'Arminio Monforte, A. De Luca, C. Gervasoni, E. Girardi, S. Lo Caputo, F Maggiolo, R. Murri, C. Mussini, M. Puoti, C. Torti

### 4. STATISTICAL AND MONITORING TEAM

A Cozzi-Lepri, I Fanti, T Formenti

### 5. PARTICIPATING PHYSICIANS AND CENTERS

M. Montroni, A. Giacometti, A Costantini, A. Riva (Ancona); U. Tirelli, F. Martellotta (Aviano-PN); G. Angarano, N. Ladisa, (Bari); F. Suter, F. Maggiolo (Bergamo); PL: Viale, G. Verucchi, B Piergentili, (Bologna); G. Carosi, G. Cristini, C. Torti, C. Minardi, D. Bertelli (Brescia); T. Quirino, C Abeli (Busto Arsizio); P.E. Manconi, P. Piano (Cagliari); J Vecchiet, K Falasca (Chieti); G Carnevale, S Lorenzotti (Cremona); F. Ghinelli, L. Sighinolfi (Ferrara); F. Leoncini, F. Mazzotta, M. Pozzi, S. Lo Caputo (Firenze); G. Pagano, G. Cassola, G Viscoli, A. Alessandrini, R. Piscopo, G Mazzarello (Genova); F. Soscia, L. Tacconi (Latina); A. Orani, R. Rossotto (Lecco); A. Chiodera, P. Castelli (Macerata); M Galli, A. Lazzarin, G. Rizzardini, I Schlacht, A. d'Arminio Monforte, AL Ridolfo, A Foschi, A Castagna, S Salpietro, S. Merli, S. Melzi, M.C. Moioli, P Cicconi, T Formenti (Milano); R. Esposito, C. Mussini (Modena); A Gori, F Sabbatini (Monza), N. Abrescia, A. Chirianni, M. De Marco, R. Viglietti, (Napoli); C. Ferrari, P. Pizzaferri (Parma); F Baldelli, B Belfiori (Perugia); G. Magnani, M.A. Ursitti (Reggio Emilia); M. Arlotti, P. Ortolani (Rimini); R. Cauda, M Andreoni, A. Antinori, G. Antonucci, P. Narciso, V Tozzi, V. Vullo, A. De Luca, M. Zaccarelli, L Gallo, R. Acinapura, P. De Longis, M.P. Trotta, A Miccoli, F. Carletti, (Roma); M.S. Mura, G Madeddu (Sassari); P. Caramello, G. Di Perri, G.C. Orofino, M Sciandra (Torino); E. Raise, F. Ebo (Venezia); G. Pellizzer, D. Buonfrate (Vicenza).

## Pre-publication history

The pre-publication history for this paper can be accessed here:

http://www.biomedcentral.com/1472-6963/12/38/prepub
